# The Bovine Hepatic Cell Line BFH12 as a Possible Model for Hepatosteatosis in Dairy Cows

**DOI:** 10.3389/fvets.2022.840202

**Published:** 2022-03-11

**Authors:** Kristin Reichelt, Anna M. Niebisch, Johannes Kacza, Axel Schoeniger, Herbert Fuhrmann

**Affiliations:** ^1^Faculty of Veterinary Medicine, Institute of Biochemistry, University of Leipzig, Leipzig, Germany; ^2^BioImaging Core Facility, Faculty of Veterinary Medicine, University of Leipzig, Leipzig, Germany

**Keywords:** bovine cell line, fatty liver (FL), lipid droplets (LDs), phospholipid, triacylglycerol (TAG), live cell imaging

## Abstract

Hepatosteatosis is a common metabolic disorder of dairy cows, especially during early lactation. Currently, there are a few models of bovine hepatic steatosis available, including primary hepatocytes, liver slices, and animal models. Studies that elucidate the influence of single fatty acids on lipid classes, fatty acid pattern, gene expression, and phenotypic changes are still limited. Hence, we investigated the suitability of the fetal bovine hepatocyte-derived cell line BFH12 as a model for hepatosteatosis. To create a steatotic environment, we treated BFH12 with stearic acid, palmitic acid, or oleic acid in non-toxic doses. Thin-layer chromatography and gas chromatography were used to analyze lipid classes and fatty acid pattern, and qPCR was used to quantify gene expression of relevant target genes. Lipid droplets were visualized with confocal laser scanning microscopy and evaluated for number and size. Treatment with oleic acid increased triglycerides, as well as lipid droplet count per cell and upregulated carnitine palmitoyl transferase 1, which correlates with findings of *in vivo* models. Oleic acid was largely incorporated into triglycerides, phospholipids, and non-esterified fatty acids. Stearic acid was found mainly in non-esterified fatty acids and triglycerides, whereas palmitic acid was mainly desaturated to palmitoleic acid. All three fatty acids downregulated stearyl-CoA-desaturase 1. In conclusion, BFH12 can acquire a steatotic phenotype by incorporating and accumulating fatty acids. Oleic acid is particularly suitable to produce hepatosteatosis. Therefore, BFH12 may be a useful *in vitro* model to study bovine hepatosteatosis and its underlying molecular mechanisms.

## Introduction

Diseases associated with parturition are gaining more and more attention in the dairy industry. Up to 50% of dairy cows show elevated triglyceride (TAG) levels in early lactation, leading to mild or severe fatty liver. This hepatosteatosis (HS) is associated with a higher risk for metabolic diseases and reproductive problems (1). The diagnosis and treatment of HS is difficult and expensive. The diagnostic gold standard is liver biopsy, while the only proven treatment is long-term intravenous infusion of glucagon (2). The main cause of HS is a negative energy balance (NEB) after parturition. With the onset of lactation, whenever dry matter intake lacks behind energy demand for subsistence and milk production, the organism massively mobilizes fat from the peripheral organs, mainly adipose tissue. Stored lipids are hydrolyzed into non-esterified fatty acids (NEFA) and distributed to other organs *via* the blood circulation, where they are processed further.

The most important organ for lipid metabolism is the liver, where NEFA are utilized in four major biochemical pathways. Firstly, this may be the oxidation to acetyl coenzyme A (Ac-CoA) *via* the beta-oxidation pathway. This leads to the formation of the ketone bodies acetoacetate and beta-hydroxybutyrate, which are subsequently released into circulation. Secondly, an oxidation of Ac-CoA to CO_2_ in the mitochondrial tricarboxylic acid cycle may occur. Thirdly, re-esterification of NEFA can form TAGs and lead to their deposition in the liver. Finally and fourthly, TAG synthesis and their packing into very low-density lipoproteins make export to extrahepatic organs possible (3). In case of massive load of fatty acids (FA), the liver tries to oxidize most of them; nevertheless, this pathway is limited by the amount of oxaloacetate in mitochondria. For unknown reasons, the assembling of very low-density lipoproteins (VLDL) is slow in ruminants (4). Only the pathways one and three remain, leading to a ketonemia and massive lipid storage in the liver (hepatosteatosis).

Excessive lipid storage is realized by formation of lipid droplets (LD) appearing close to the endoplasmic reticulum (5). LDs range in size from 0.1 μm to 150 μm, and they consist of a phospholipid monolayer surrounding a hydrophobic core with neutral lipids, mainly TAG and cholesterol esters (ChE) (5). The formation of LDs in hepatocytes is a hallmark of HS (6). Fatty acid overload *in vitro* has been reviewed recently (7). According to that review, monounsaturated FAs allow higher lipid accumulation, whereas saturated FAs are more lipotoxic. In HepG2, palmitate and stearate, but not monounsaturated palmitoleate or oleate, generate stress in the endoplasmic reticulum. Until today, only primary bovine hepatocytes and non-bovine cell lines, such as mouse, rat, or human, were used to investigate hepatic metabolism. To gain a deeper understanding of the underlying cellular mechanisms of HS, investigating HS in dairy cows is necessary. For that purpose, several cell culture models of HS have been developed. However, to our knowledge, no model from a bovine cell line has been reported. Gleich et al. (8, 9) from our workgroup created and established the bovine cell line BFH12 (RRID:CVCL_JQ51), which shares many metabolic properties with primary hepatocytes, like epithelial growth and numerous cytoplasmatic granules. BFH12 expresses important enzymes and carrier proteins; stores glycogen and lipids; and produces urea, lactate, and triglycerides. Consequently, BFH12 may be an appropriate model to study bovine HS.

The current study aims to assess BFH12 as a model of bovine HS for further clinical and pharmaceutical research. Therefore, cells were treated with stearic (SA), palmitic (PA), or oleic acid (OA) in non-toxic concentrations to analyze changes in fatty acid pattern. Furthermore, LDs as hallmark of HS were evaluated with confocal laser scanning microscopy. Additionally, gene expression of key enzymes in lipid metabolism was measured before and after treatment.

## Materials and Methods

### Cell Culture

BFH12 was cultured in 75-cm^3^ cell culture flasks with Williams' Medium E (WME; BIO&SELL, Nuremberg, Germany) containing 600 mg/l glucose, supplemented with 5% heat-inactivated fetal bovine serum (FBS superior, Biochrom/Merck, Berlin, Germany), 1% penicillin/streptomycin (Biochrom/Merck, Berlin, Germany), 2 mM L-alanyl-L-glutamine (Biochrom/Merck, Berlin, Germany), 100 nM dexamethasone (Sigma-Aldrich, Taufkirchen, Germany), and 0.2 U/ml insulin (Sigma-Aldrich, Taufkirchen, Germany). Cells were incubated at a density of 5 × 10^3^ cells/cm^2^ at 37°C and 5% CO_2_ in a humidified atmosphere. The medium was changed after 3 days. Cell detachment for subcultivation or harvest was performed on day 7 by treatment with trypsin/EDTA (0.5/0.2%; Biochrom/Merck, Berlin, Germany) solution for 8 min at 37°C. Cell number and viability were checked microscopically (Zeiss Axiovert, Oberkochen, Germany, ×100) with an improved NEUBAUER chamber with trypan blue (Sigma-Aldrich, St. Louis, MO, USA).

### Pilot Studies

The aim of the pilot studies was to determine for the BFH12 non-toxic, but steatotic concentrations, of SA, PA, and OA. We used the 3-(4,5-dimethylthiazol-2-yl)-2,5-diphenyl tetrazolium bromide assay (MTT, Org. Chemie Uni Greifswald, Germany) to check cell viability and Oil Red O staining to visualize LDs. As a base medium, we used WME 5% containing 0.75 mM cholesterol, 0.56 mM TAG, and 0.059 mM NEFA. This includes 39.6 μM PA, 6.6 μM SA, and 11.1 μM OA, originating from the heat-inactivated FBS. During the pilot study, we added increasing concentrations of each fatty acid:

- 5.4–60.4 μM PA (Matreya, PA, USA)
- 18.4–103.4 μM SA (Matreya, PA, USA)
- 23.9–288.9 μM OA (Sigma-Aldrich, Taufkirchen, Germany).

The MTT assay is based on an active metabolism and not only on an intact cell membrane. Viable cells with active metabolism convert MTT into a purple-colored formazan product with an absorbance maximum near 570 nm. When cells die, they are unable to convert MTT into formazan; thus, color formation serves as a marker of viable cells (10). Oil Red O staining was performed following the protocol from Gleich et al. (8) and used to determine the concentration where LDs are visible.

From these experiments, the final FA concentrations used during the following experiments were derived:

PA:            **60** **μM PA**,    6.6 μM SA,    11.1 μM OA
SA:            39.6 μM PA,   **50** **μM SA**,   11.1 μM OA
OA:           39.6 μM PA    6.6 μM SA    **100** **μM OA**
Control:    39.6 μM PA,   6.6 μM SA,   11.1 μM OA

**Figure 1:**
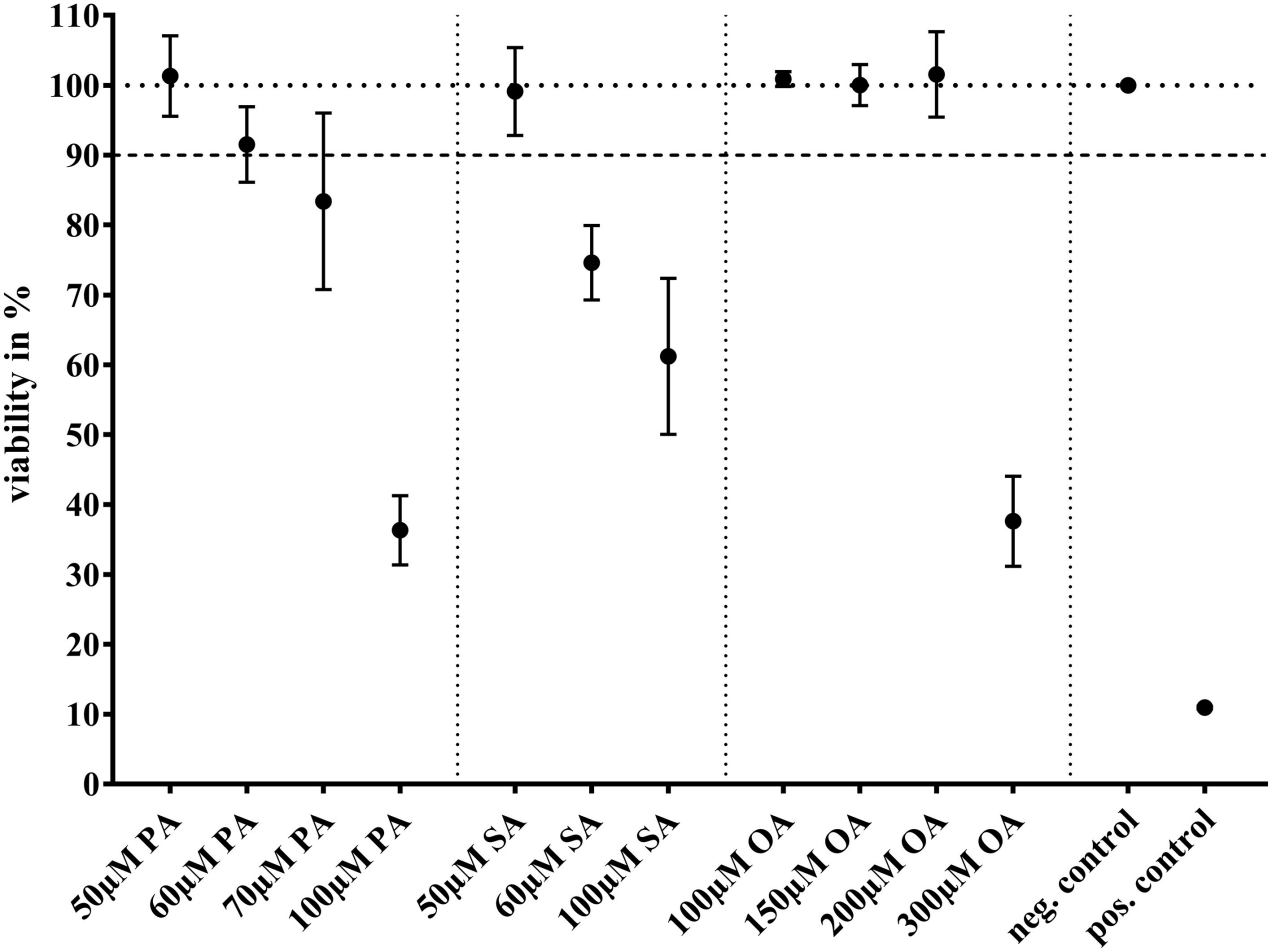
MTT assay showing viability (in %) after treatment with different concentrations of palmitic acid (PA; *N* = 3; *n* = 6), stearic acid (SA; *N* = 3; *n* = 3), or oleic acid (OA; *N* = 3; *n* = 3). For negative control, cells were treated with base medium + 0.1 % ethanol and for positive control with Triton X (5 mg/ml in PBS). A 90% viability was accepted for further experiments. Dots represent arithmetic mean and whiskers standard deviation.

These concentrations found to be not yet toxic to BFH12. With higher concentrations of the single FAs, the growth of BFH12 was significantly depressed (Figure 1). Cells would have tolerated higher margins of OA, but we opted for 100 μM due to its limited solubility.

### Treatment With Palmitic, Stearic, and Oleic Acid

All three FAs were solubilized in ethanol to prepare stock solutions (1,000×) that were stored in the dark at −20°C. After 3 days of cultivation, cells grew close to confluence, and at this time, BFH12 was treated. Shortly before treatment, the stock solutions and WME 5% were slowly heated to 40°C. Stock solution was added to base medium (1:1,000) under constant stirring. Final ethanol concentration was 0.1% and chosen as control. After 24 h of incubation, cells were harvested and/or prepared for further analysis.

### Total FA, Lipid Class Composition, and Fatty Acid Pattern

To analyze the FA composition with gas chromatography, 1 × 10^7^ cells were processed in three sequential steps: extraction of lipids, separation of the lipid classes by thin-layer chromatography, and methylation of the FAs following the protocol from Adolph et al. (11).

In addition to the lipid class composition and fatty acid pattern, the methylene bridge index (MBI) and different desaturase activity indices were calculated. The MBI expresses the mean number of *bis*-allylic methylene bridge positions per FA, as a measure of oxidizability of FA. It was calculated by multiplying the number of *bis*-allylic methylene bridges in each FA species by its respective mole fraction and summed up for all unsaturated FAs (12).

Desaturase activity indices were calculated using the product/precursor ratio of individual or grouped FAs according to the following notation:


$$\begin{array}{l} \Delta 9-\text{desaturase }=\;\frac{\text{C}14:1\text{n}5+\text{C}16:1\text{n}7+\text{C}18:1\text{n}9}{\text{C}14:0+\text{C}16:0+\text{C}18:0}\\ \Delta 5-\text{desaturase }=\frac{\text{C}20:4\text{n}6}{\text{C}20:3\text{n}6} \end{array}$$

### Statistics for FA Data

Statistical analysis was performed with IBM SPSS 27. Data was checked for outliers by Grubbs' test (*p* < 0.05) with GraphPad Prism (version. 7.05); outliers were found in all lipid classes and removed from further analysis. Afterwards, data were checked for normal distribution by Shapiro–Wilk test (*p* < 0.05) and for homogeneity of variances with Brown–Forsythe test (*p* < 0.05). A one-way analysis of variance (ANOVA) was performed to check for significant effects of the FA added. All datasets that met at least homogeneity of variance were analyzed with one-way ANOVA, as this test is robust to violations of the normal distribution (13). As *post-hoc* test, Bonferroni test was used. Some data also violated the assumption of homogeneity of variances. For these datasets, we used Welch's ANOVA with Dunnett T3 as *post*-*hoc* test. Differences were considered significant for *p*-values < 0.05.

### Fluorescent Staining

Cells were seeded on ibidi® μ-Slide VI^0.4^ flow chambers (ibidi GmbH, Gräfelfing, Germany) at a density of 3 × 10^3^ cells/channel and treated as described above. After 24 h of treatment, LDs were stained with LipidSpot 610 (0.5 × diluted in WME; Biotium, Biotrend, Köln, Germany), and nuclei were stained with DAPI (1 μg/ml; Roth, Karlsruhe, Germany). After staining, cells were washed with PBS, and 100 μl WME were added to each μ-slide channel.

### Confocal Laser Scanning Microscopy

**Figure 2:**
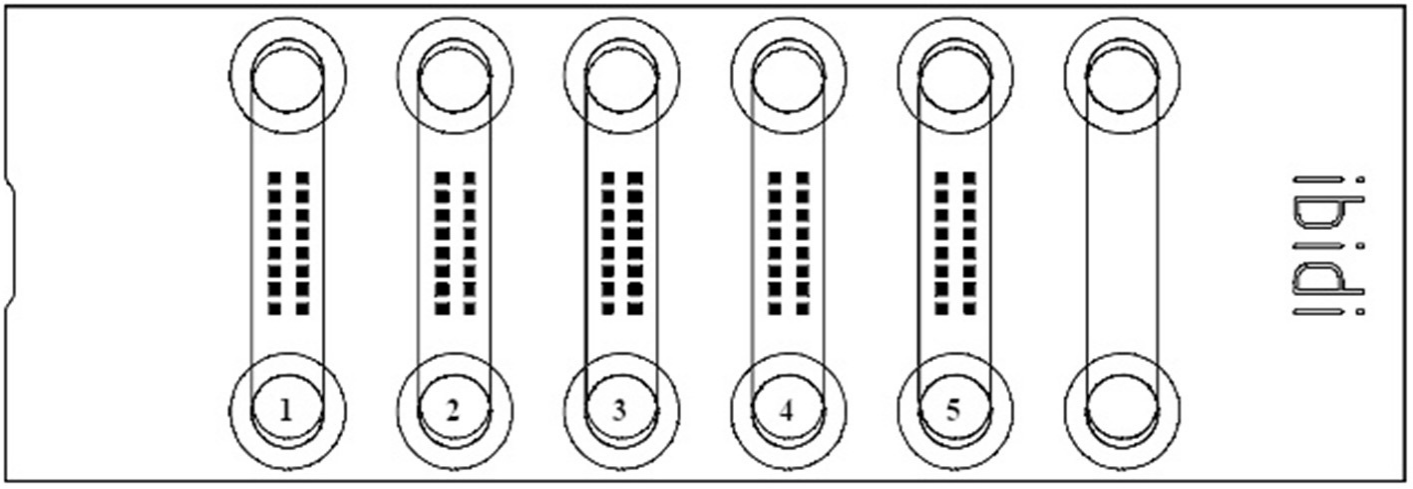
Schemata of an ibidi® μ-Slide VI^0.4^ flow chamber with the rectangular grid of 16 defined areas [filled square (■); 184.5 × 184.5 μm], where images were captured for further analysis of lipid droplets. The different treatments for each μ-slide are shown: (1) 100 μM oleic acid; (2) base medium; (3) 50 μM stearic acid; (4) control; and (5) 60 μM palmitic acid.

Cells were imaged with the confocal laser scanning microscope (CLSM) Leica TCS SP8 (Leica Microsystems, Mannheim, Germany). Confocal settings were as follows: scan speed 600 Hz, pinhole 1 Airy unit, 2× line averaging, voxel size 68.5 × 68.5 × 375 nm, excitation wavelength 405 nm (DAPI) and 633 nm (LipidSpot 610), detection range 407–554 nm (DAPI) and 638–714 nm (LipidSpot 610), and refraction index for lens and embedding media was 1.33. Using a ×63/1.20 water objective and the Leica stage navigator software (LAS X 3.5.5), image stacks (9 or 13 layers each) were automatically scanned at 16 positions in each μ-slide channel. To achieve a random distribution of cells in the images (184.5 × 184.5 μm), the scan positions were arranged in a defined 2 × 8 matrix centered in each μ-slide channel (Figure 2). Prior to recording images, a focus map was created for each μ-slide channel. This map was based on the maximum intensity of the LipidSpot 610 fluorescence nearby but not overlapping, with the defined scan positions. Focus map positions were also used to determine the transmission of the 633-nm laser just below the signal saturation of the hybrid detector. Using the focus map image, stacks were automatically recorded at their appropriate *z*-position to ensure that each stack contained the layer of maximum intensity of the LipidSpot 610 fluorescence. Finally, CLSM raw data was processed by LAS X automatic dye separation.

### Deconvolution

In order to reduce image aberrations, deconvolution of CLSM data was performed using Huygens professional, version 20.10 (SVI, Hilversum, Netherlands). Microscopic parameters were used according to image meta data, as mentioned in the *Confocal Laser Scanning Microscopy* section. A theoretical point spread function was applied, and the following parameters were set for batch processing of image stacks: maximal iterations = 40, quality change threshold = 0.1, signal-to-noise ratio = 12, and relative background = 0. After deconvolution, image stacks were cropped by selecting the layer showing the highest intensity of the LipidSpot 610 fluorescence using the software's “plot intensity flux function.” The selected layers were saved for further image analysis.

### Image Analysis

**Figure 3:**
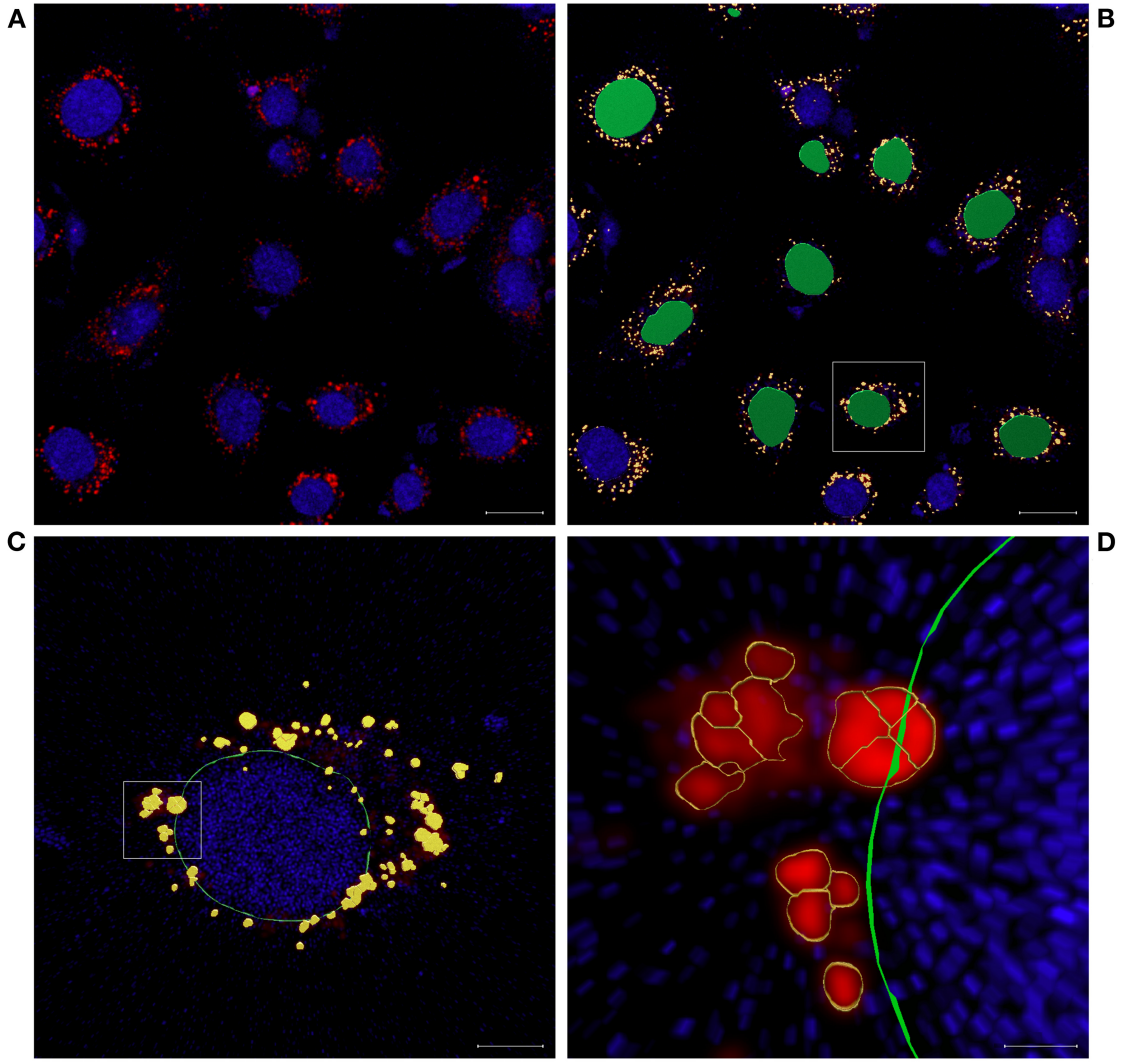
BFH12 treated with 100 μM oleic acid. **(A)** All labeled cells in one of the defined areas on the flow chamber, nuclei stained with DAPI (blue), and lipid droplets (LD) stained with LipidSpot 610 (red). **(B)** same image as (A) with nuclei surfaces (green) and LD surfaces (yellow) as they are present after image segmentation and nuclei that do not meet criteria for analyses (blue). **(C)** Enlargement of (B); nucleus is marked with a green outline for better contrast to quantified LD surfaces (yellow). **(D)** Enlargement of (C); irregular-shaped LDs and cluster of small LDs; nucleus is marked with a green outline, and LDs are marked with yellow outline; scale bar: **(A)** and **(B)** = 20 μm; **(C)** = 5 μm; **(D)** = 1 μm.

Images analysis was done using the software Imaris, version 9.7.2 (Oxford Instruments, Abingdon, UK). First, image segmentation was applied to convert voxel-based data of CLSM images into individual and quantifiable surface objects. In order to achieve uniform segmentation for all images, a template was created, containing the following creation parameters: surface grain size = 0.4 μm, diameters of largest sphere = 1.0 μm, manual threshold value = 22.74, seed point diameter = 0.42 μm, quality > 7.94, and filter surface > 12.8 voxels. Surface objects of one to eight cells of each image were selected for quantification of number and projected area (number of voxels/surface object) according to the following criteria: surfaces did not touch the image borders, surfaces of cell nuclei showed no fractal geometries or inclusions of other surfaces, clear spatial correlation between nucleus and associated LDs was given, and there was an apparent completeness of LDs associated with a nucleus (specific problem of near-border cells) (Figure 3). Voxel counts were used to calculate surface areas of LDs and nuclei.

### Statistics for CLSM

Statistical analysis was performed with IBM SPSS Statistics 27. LD surface areas and LDs per cell were checked for normal distribution (Shapiro–Wilk test) and homogeneity of variances (Levene's test). Since both were not given, we decided to use Welch's ANOVA. This statistical test is robust to violations of normal distribution and homogeneity of variances (14). As *post hoc* test, Games-Howell test was administered. Data was also checked for correlation between the size of the nucleus and the number of LDs per cell, with Spearman's correlation test. We found a low positive linear correlation (*r* = 0.217; *p* < 0.001). Therefore, we calculated the ratio of LDs per cell to the size of the nucleus surface area (LD ratio = LDs / 100 μm^2^ nucleus surface area). This ratio was analyzed similar to the data of the LD surface areas. Again, there was no normal distribution and no homogeneity of variance, so we applied Welch's ANOVA. For all tests, *p* < 0.05 was set as significance level.

### Primer Design and Testing

Species-specific primers were designed based on nucleotide sequences available in the NCBI database using Primer-BLAST (https://www.ncbi.nlm.nih.gov/tools/primer-blast/ last accessed 29th January 2019) and are shown in Table 1. Different reference genes were tested, and the most stable combination for the BFH12 cell line was determined using NormFinder (15). Primers were synthesized and HPLC purified by Metabion (Planegg, Germany).

**Table 1:** Primers of the housekeeping genes (GAPDH, glyceraldehyde 3-phosphate dehydrogenase; SDHA, succinate dehydrogenase complex flavoprotein subunit A) and primers of fatty acid metabolism-related genes.

**Gene name**		**Sequence of primers (5^**′**^-3^**′**^)**	**Length of amplicon (bp)**	**GenBank^**TM**^ accession number**	**Annealing temperature**
**Housekeeping genes**					
GAPDH		fwd GAAGGTCGGAGTGAACGGAT rev GTGGGTGGAATCATACTGGAA	148	NM_001034034.2	60°C
SDHA		fwd ACCTGATGCTTTGTGCTCTGC rev TTCTTCTGCTGCCCCTGGA	139	NM_174178.2	61°C
**Genes of interest**					
ACADL	a	fwd CATGTTTGAGGAAACCAGGG rev GCTTCACCGTCTGTAAATCTG	80	NM_001076936.1	63°C
CPT1A	a	fwd CAAATCATGCACTGTTGACC rev AGAGACAAGCCCAAATAGGTT	100	NM_001304989.1	63°C
GPAM	b	fwd AGCCAAAGTCAGAAACCTGT rev ACTGGGTCTTGAGGGAAGTA	81	NM_001012282.1	63°C
SCD1	b	fwd CCAGAGGAGGTACTACAAAC rev CGTTTCATCCCACAGATACC	85	NM_173959.4	60°C
ApoB	c	fwd GCACTGTCTCCATTACCTCTAC rev CGTGGCAAAGTCCTCATACTTC	131	XM_024999521.1	62°C
PLN5	c	fwd ACTTTTGACCCGATGGGACC rev AGTAGTGCTGACGCATAGCC	93	NM_001101136.1	60°C
SREBP1c	d	fwd GCTACCGCTCTTCCATCAAT rev GGCAGATTTATTCAACTTGGC	83	NM_001113302.1	63°C

a *Fatty acid oxidation pathway: ACADL, acyl-CoA dehydrogenase long chain; CPT1A, carnitine palmitoyl transferase 1*.

b *Triglyceride synthesis pathway: GPAM, glycerol-3-phosphate acyltransferase mitochondrial; SCD1, stearoyl-CoA desaturase 1*.

c *VLDL metabolism: ApoB, apolipoprotein B100; PLN5, perilipin 5*.

d *Fatty acid de novo synthesis: SREBP1c, sterol regulatory element-binding protein-1c*.

Primers were tested according to the MIQE Guidelines (16), including a qualitative PCR using FastGene Optima Hotstart Ready Mix (Nippon Genetics, Dueren, Germany), which was carried out according to manufacturer's instructions. Amplicons were analyzed by electrophoresis in 10 % Tris acetate EDTA-buffer at 60 V for 50 min on a 1.5 % agarose gel containing GelRed® (Biotium, Fremont, CA, USA). Documentation was carried out using a GeneGenius Bio Imaging System (Syngene, Cambridge, UK), GeneSnap (Syngene, Cambridge, UK, version 7.12.06), and GeneTools software (Syngene, Cambridge, UK, version 4.03.05.0). Primer efficiencies were tested under experimental conditions and accepted in a range from 0.7 to 1.1. Finally, seven genes of interest and two reference genes were retained in the study.

### qPCR

Cells were treated and obtained as described above. For total RNA extraction, 1 × 10^6^ cells were used, which was performed with NucleoSpin RNA plus (Macherey-Nagel, Düren, Germany) according to the manufacturer's instructions. Genomic DNA was removed from samples using DNase I (Thermo Fisher Scientific, EN0521, Waltham, MA, USA). RNA concentration was analyzed with the UV-spectrophotometer Genesys 10 (Thermo Fisher Scientific, Waltham, MA, USA) and RNA integrity number (RIN) ranked between 9.5 and 10 for all samples, which was determined with an Agilent 2100 Bioanalyzer. RNA was stored at −80°C until further utilization. Afterwards, cDNA synthesis was performed using ProtoScript II kit (New England Biolabs, Frankfurt am Main, Germany). For qPCR, the SensiMix™ SYBR® No-ROX Kit from Bioline was used according to manufacturer's instructions. qPCR was performed with the PCR thermo cycler Rotor-Gene 6000 (Qiagen, Hilden, Germany). The reaction mix was heated to 95°C for 10 min to activate polymerase. This was followed by 45 cycles at the following settings: 95°C for 15 s (melting), 60°C for 30 s (annealing), and 72°C for 15 s (elongation). A melting curve was recorded between 55°C and 99°C. The experiment was performed three times, each time with three technical replicates for each gene.

#### Data Analysis and Statistics for qPCR Data

Data from Q-Rex software (Qiagen, Hilden, Germany, 2013) was analyzed by relative expression software tool (REST, version 2.0.13; Qiagen, Hilden, Germany) (17). We used the REST-RG mode, which is designed to directly enter the Rotor-Gene's comparative quantitation outputs: take-off point and amplification. Both parameters are calculated for each sample individually. Take-off point describes where a reaction first deviates from the background, and amplification means the reaction efficiency for a single sample. Software compares treated with control samples and calculates expression, standard error, confidence interval, and *P*-value of a hypothesis test, based on 10,000 random reallocations of samples and controls. *p*-values < 0.05 are considered to be significant.

## Results

### Total FA and Lipid Classes in BFH12

Total amounts of FA increased after treatment with OA (Table 2).

**Table 2:** Total amount of fatty acids (FA) after supplementation with palmitic acid (PA), stearic acid (SA), or oleic acid (OA).

	**PA**	**SA**	**OA**	**Control**
	**(*n* = 5)**	**(*n* = 5)**	**(*n* = 5)**	**(*n* = 5)**
**FA in nmol/10**^**7**^ **cells**				
	1,265.12 ± 98.21	1,189.94 ± 48.22	1,623.87 ± 93.1	1,136.1 ± 100.65
*P*-value	>0.99	>0.99	0.031	

*Analysis was performed in five independent experiments. Data is presented as mean ± SEM. Significant differences in FA content between control and treated cells are identified by p-values ≤ 0.05*.

**Figure 4:**
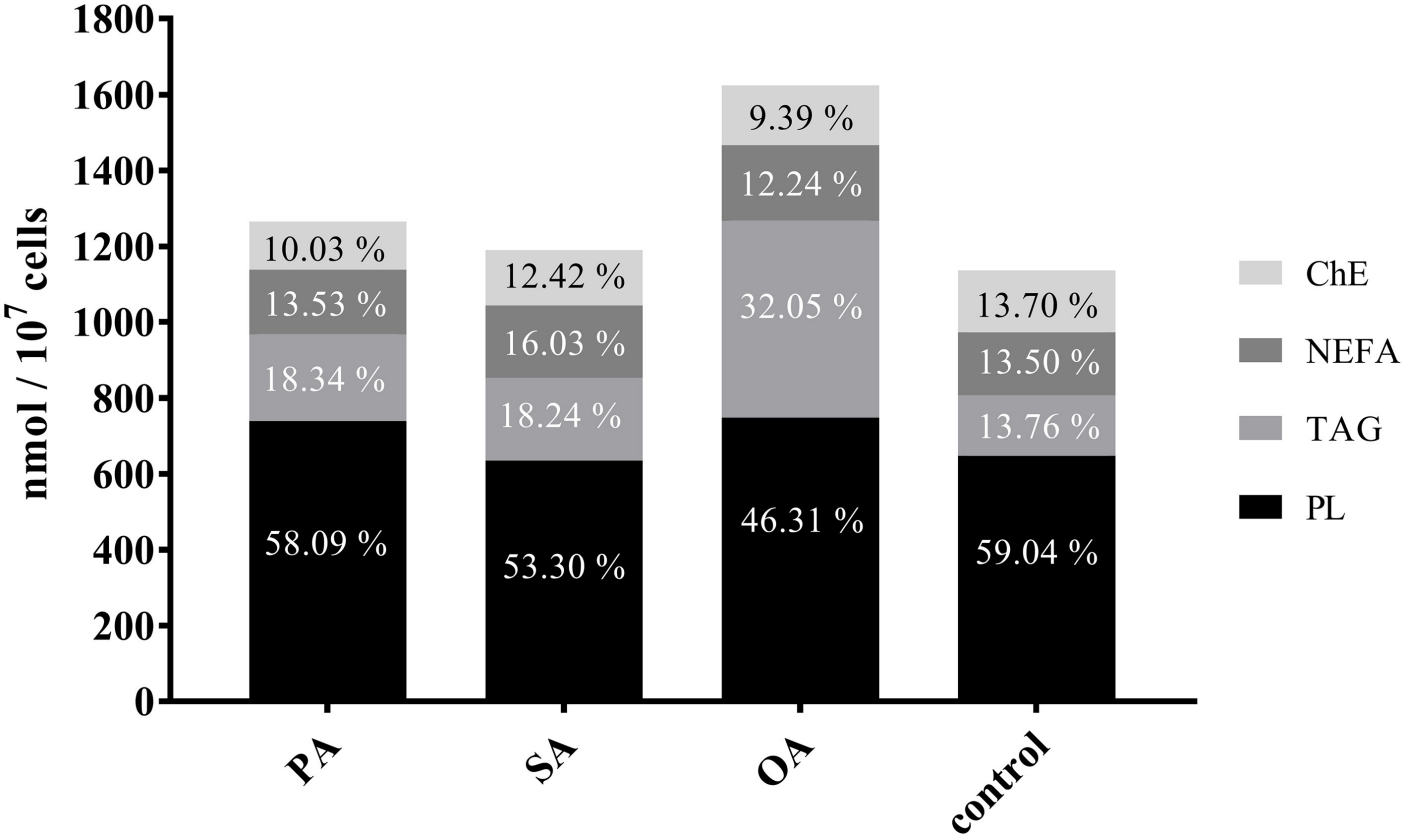
Proportion of lipid classes in control and treated cells. Bars show the total fatty acids in nmol FAs/10^7^ cells. For comparability, the proportions of the lipid classes (ChE, cholesterol esters; NEFA, non-esterified fatty acids; TAG, triacylglycerides; PL, phospholipids) are indicated. PA, palmitic acid; SA, stearic acid; OA, oleic acid.

The percentage of lipid classes shifted significantly after the different treatments. The proportion of phospholipids decreased by 13% after treatment with OA and by about 5% after the addition of SA. After addition of OA, the proportion of TAG increased nearly threefold, while there was only a 5% increase after the treatment with SA and PA, respectively. An increase in NEFA was noticeable after addition of SA only. ChE slightly decreased with all treatments (Figure 4).

### FA Composition of Lipid Classes in BFH12

In all experiments, a significant increase in the added FAs was observed. In addition, various longer- and shorter-chain FAs also rose. The focus of the evaluation is on PLs as the main component of membranes, which also surround the LDs, and on TAG, as the main storage form of FAs. Specific changes in fatty acid pattern of NEFAs and ChEs are presented partly.

### Phospholipids

Treatment with PA increased C16:1n7 as desaturation product and total n3 FA. C14:0 decreased significantly. Supplementation with SA led to a decline in C16:0 and C14:0. C 18:1n7 was significantly lower than in the control group. OA led to a rise in total n9 FA, whereas C16:1n7 and C14:0 dropped. The Δ5-desaturase index nearly doubled (Table 3).

**Table 3:** Fatty acid pattern of PL.

	**PA**	**SA**	**OA**	**Control**
	**(*n* = 5)**	**(*n* = 5)**	**(*n* = 5)**	**(*n* = 5)**
**FA in nmol/10**^**7**^ **cells**				
**Total FA**	739.22 ± 74.11	634.58 ± 27.85	748.12 ± 36.54	647.83 ± 71.89
*p*-value	>0.99	>0.99	>0.99	
**Saturated FA**	277.71 ± 8.84^*^	263.3 ± 9.54	195.0 ± 16.79	272.4 ± 28.57
*p*-value	>0.99	>0.99	0.052	
**MUFA**				
total n7	127.29 ± 19.82	43.85 ± 3.43	56.00 ± 5.85	85.91 ± 14.99
*p*-value	0.224	0.208	0.720	
total n9	146.2 ± 15.4	195 ± 11.04	367.1 ± 23.2	137.7 ± 14.67
*p*-value	>0.99	0.163	<0.001	
**PUFA**				
total n3	51.73 ± 2.89	43.04 ± 1.72	33.23 ± 1.64	41.72 ± 2.07
*p-*value	0.027	>0.99	0.076	
total n6	60.24 ± 3.53	51.59 ± 1.97	42.88 ± 1.34	49.34 ± 3.62
*p*-value	0.084	>0.99	0.727	
**Single FA**				
C14:0	9.5 ± 1.9	4.6 ± 0.54	8.30 ± 0.12^*^	17.59 ± 1.45
*p*-value	0.002	<0.001	0.001	
C16:0	214.6 ± 19.06	108.8 ± 9.74	129.7 ± 15.55	188.1 ± 21.28
*p*-value	>0.99	0.027	0.163	
C16:1n7	58.03 ± 5.91	13.38 ± 0.99	6.72 ± 0.47	24.89 ± 3.66
*p*-value	0.012	0.135	0.031	
C18:0	67.65 ± 4.41	141.3 ± 5.5	45.7 ± 1.59	55.81 ± 5.96
*p*-value	0.557	<0.001	0.88	
C18:1n9	120.4 ± 12.65	176.9 ± 9.68	334.7 ± 21.99	116.6 ± 12.7
*p*-value	>0.99	0.070	<0.001	
C 18:1n7	50.69 ± 4.65^*^	28.12 ± 2.61	46.7 ± 5.33	56.73 ± 10.71
*p*-value	>0.99	0.047	>0.99	
**MBI**	399.1 ± 23.65	334.8 ± 7.37	280.7 ± 9.95	330.6 ± 21.53
*p*-value	0.073	>0.99	0.34	
***Δ*** **9-desaturase**	0.61 ± 0.02	0.75 ± 0.03	1.94 ± 0.24	0.546 ± 0.02
*p*-value	0.202	0.003	0.017	
***Δ*** **5-desaturase**	5.43 ± 0.24	4.86 ± 0.04	10.26 ± 0.29	5.804 ± 0.21^*^
*p*-value	>0.99	0.057	<0.001	

*Analysis was performed in five independent experiments. Data are presented as mean ± SEM. Significant differences in fatty acid (FA) content between control and treated cells are identified by p-values ≤ 0.05*.

* *One outlier removed by Grubbs' test. PA, palmitic acid; SA, stearic acid; OA, oleic acid; MBI, methylene bridge index*.

### Triglycerides

Supplementation of PA led to an increase of its desaturation products (C16:1n7 and C16:1n9). C14:0 and n7 FA augmented significantly, as well as total n9 FA. An expected finding was the surge of total saturated FAs by about 50 nmol.

After treatment with SA, SA itself and OA levels rose. Degradation products of SA remained unaffected, while total n9 FA increased.

OA supplementation led to an enormous rise of C18:1n9. The β-oxidation products C16:1n9 and also C14:0 augmented significantly. An increase is also seen in n6 and n3 FA. The MBI and the Δ9-desaturase index rose significantly after treatment with OA (Table 4).

**Table 4:** Fatty acid pattern of TAG.

	**PA**	**SA**	**OA**	**Control**
	**(*n* = 5)**	**(*n* = 5)**	**(*n* = 5)**	**(*n* = 5)**
**FA in nmol/10**^**7**^ **cells**				
**Total FA**	229.09 ± 15.05	218.3 ± 22.01	518.68 ± 29.03	159.12 ± 5.77
*p-*value	0.032	0.210	0.001	
**Saturated FA**	155.4 ± 13.87	141.6 ± 11.28	11.8 ± 15.02	102.2 ± 4.09
*p-*value	0.035	0.192	>0.99	
**MUFA**				
Total n7	19.52 ± 2.36	8.06 ± 0.24^*^	49.42 ± 9.20	3.84 ± 0.4
*p-*value	0.010	<0.001	0.033	
total n9	17.48 ± 2.34	39.05 ± 2.43^*^	301.1 ± 14.36	4.12 ± 0.49
*p-*value	0.017	0.02	<0.001	
**PUFA**				
Total n3	2.25 ± 0.31	1.98 ± 0.75	4.39 ± 0.4	1.55 ± 0.14
*p-*value	>0.99	>0.99	0.003	
Total n6	2.92 ± 0.38	2.88 ± 0.51	8.34 ± 0.66	1.21 ± 0.15
*p-*value	0.118	0.134	<0.001	
**Single FA**				
C14:0	5.06 ± 0.76	3.096 ± 0.55	5.73 ± 0.43	1.97 ± 0.297
*p-*value	0.006	0.964	0.001	
C16:0	138.97 ± 12.45	89.79 ± 8.94	93.17 ± 13.66	94.15 ± 3.44
*p-*value	0.046	>0.99	>0.99	
C16:1n9	1.7 ± 0.29	1.49 ± 0.43	9.69 ± 0.61	0.14 ± 0.07
*p-*value	0.021	0.028	<0.001	
C16:1n7	7.94 ± 1.3	2.15 ± 0.13^*^	3.99 ± 0.79^*^	0.94 ± 0.03^*^
*p-*value	0.025	0.008	0.110	
C18:0	7.29 ± 1.21	43.49 ± 10.78	5.48 ± 0.71	1.42 ± 0.49
*p-*value	0.026	0.072	0.011	
C18:1n9	13.32 ± 1.92	35.06 ± 2.16^*^	279.03 ± 13.29	2.92 ± 0.32
*p-*value	0.022	0.002	<0.001	
C 18:1n7	9.91 ± 1.62	5.3 ± 0.22	34.898 ± 8.8	2.11 ± 0.29
*p-*value	0.033	<0.001	0.083	
C 20:1n9	0.54 ± 0.01^*^	0.81 ± 0.05^*^	8.31 ± 0.45	n.d.
*p-*value	<0.001	0.002	<0.001	
**MBI**	25.09 ± 2.37	24.14 ± 5.43	57.17 ± 6.17	22.04 ± 0.76
*p-*value	0.766	0.998	0.019	
***Δ*** **9-desaturase**	0.15 ± 0.014	0.22 ± 0.05	2.48 ± 0.20^*^	0.053 ± 0.001^*^
*p-*value	0.014	0.130	0.005	

*Analysis was performed in five independent experiments. Data is presented as mean ± SEM. Significant differences in fatty acid (FA) content between control and treated cells are identified by p-values ≤ 0.05*.

* *One outlier removed by Grubbs' test. n.d., not detectable; PA, palmitic acid; SA, stearic acid; OA, oleic acid; MBI, methylene bridge index*.

### Cholesterol Esters

Fatty acid pattern of ChE (data not shown) changed after treatment with OA only. OA itself rose as well as vaccenic acid (C18:1trans11), a FA that is derived from linolenic acid. Over all treatments, PA was the predominant FA in ChE.

### NEFA

The addition of PA led to a significant increase in total n7 and total n3 FA. Interestingly, PA itself and, in addition, also total saturated FA did not change significantly in NEFA.

Treatment with SA led to an increase of C18:0 itself and a rise of the desaturation product (C18:1n9). Saturated FA remained nearly unaffected.

OA supplementation caused an increase of OA and total n9 FA as expected (Table 5).

**Table 5:** Fatty acid pattern of NEFA.

	**PA**	**SA**	**OA**	**Control**
	**(*n* = 5)**	**(*n* = 5)**	**(*n* = 5)**	**(*n* = 5)**
**FA in nmol/10**^**7**^ **cells**				
**Total FA**	170.04 ± 9.49	190.54 ± 10.00	199.54 ± 16.36	166.21 ± 16.72
*p*-value	>0.99	>0.99	0.610	
**Saturated FA**	109.97 ± 5.98	120.98 ± 5.54	103.57 ± 9.96^*^	108.09 ± 9.22
*p*-value	>0.99	>0.99	>0.99	
**MUFA**				
Total n7	11.89 ± 1.76	6.19 ± 1.09	3.79 ± 1.08	4.7 ± 1.02
*p*-value	0.006	>0.99	>0.99	
Total n9	13.41 ± 1.9	27.66 ± 3.32	60.74 ± 11.89	6.7 ± 1.61
*p*-value	0.132	0.007	0.042	
**PUFA**				
Total n3	3.57 ± 0.46	2.88 ± 0.397	2.68 ± 0.29	1.39 ± 0.435
*p*-value	0.008	0.108	0.218	
Total n6	3.56 ± 0.68	3.44 ± 0.42	3.1 ± 0.35	1.59 ± 0.18
*p*-value	0.04	0.06	0.18	
**Single FA**				
C16:0	87.23 ± 1.19^*^	73.45 ± 4.48	76.18 ± 13.04	91.37 ± 8.39
*p*-value	>0.99	0.899	>0.99	
C18:0	10.77 ± 1.90	32.18 ± 2.43	6.02 ± 0.39	7.27 ± 0.96
*p*-value	0.536	0.003	0.775	
C18:1n9	10.63 ± 1.67	24.69 ± 3.12	56.94 ± 11.57	5.24 ± 1.39
*p*-value	0.178	0.008	0.045	
C 18:1n7	6.25 ± 0.92	4.09 ± 0.87	4.46 ± 0.47	3.09 ± 0.76
*p*-value	0.171	>0.99	>0.99	
**MBI**	30.89 ± 2.69	30.78 ± 0.85^*^	28.58 ± 3.06	24.19 ± 3.57
*p*-value	0.681	0.84	>0.99	
***Δ*** **9-desaturase**	0.16 ± 0.02	0.26 ± 0.01^*^	0.51 ± 0.06^*^	0.076 ± 0.01
*p*-value	0.041	<0.001	0.019	

*Analysis was performed in five independent experiments. Data are presented as mean ± SEM. Significant differences in fatty acid (FA) content between control and treated cells are identified by p-values ≤ 0.05*.

* *One outlier removed by Grubbs' test. PA, palmitic acid; SA, stearic acid; OA, oleic acid; MBI, methylene bridge index*.

### Lipid Droplets

**Figure 5:**
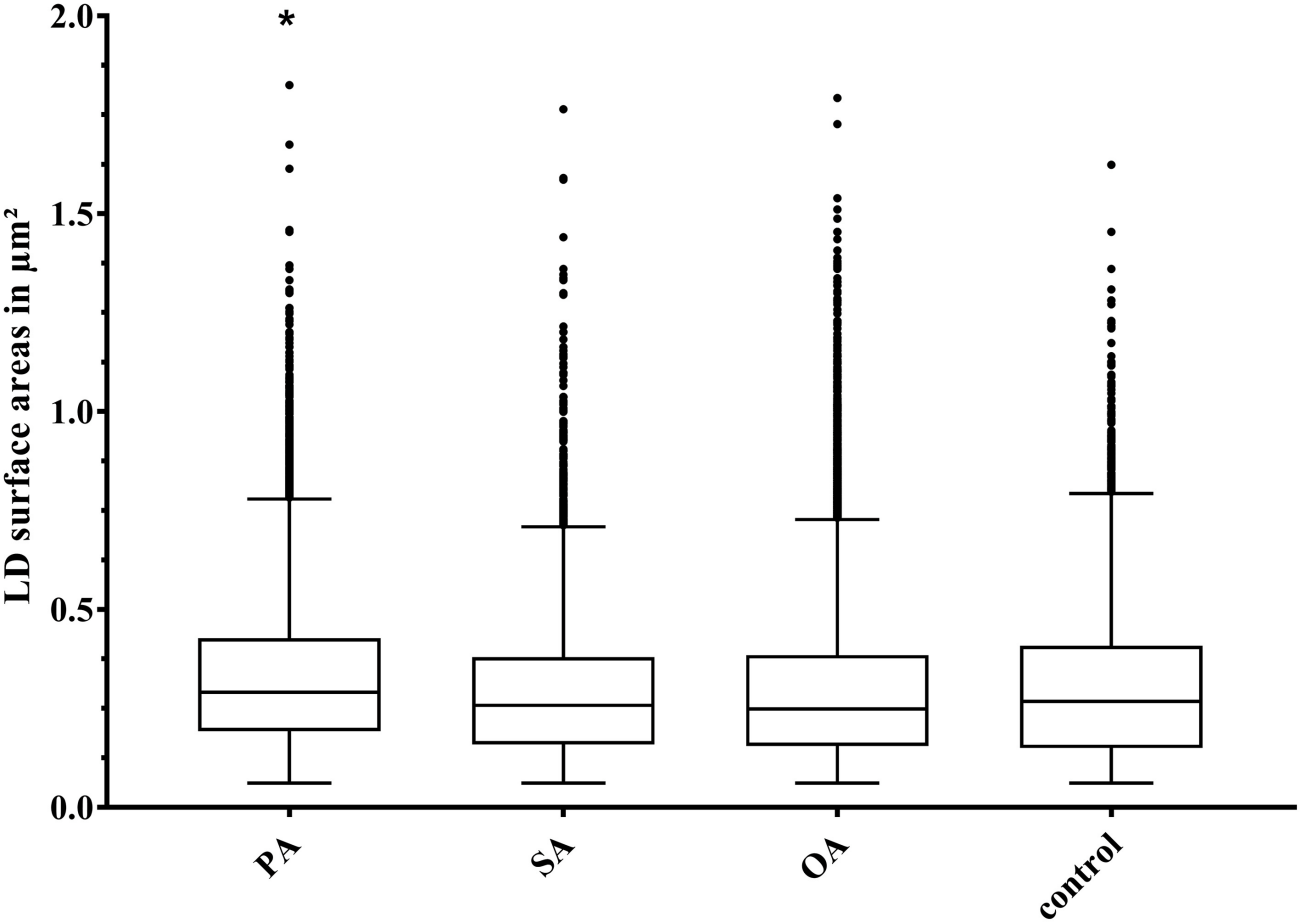
Boxplot (median; 25th to 75th percentiles; min to max) of lipid droplet (LD) surface areas (in μm^2^) after different treatments. Asterisks significant difference between treatment and control (*p* < 0.05). Dots indicate outliers; PA, palmitic acid (*N* = 9,584); SA, stearic acid (*N* = 5,008); OA, oleic acid (*N* = 18,215); control (*N* = 4,617); *N*, the number of evaluated LDs.

The size of LD surface areas ranged from 0.06 to 1.83 μm^2^ (Figure 5). The lower limit of the LD surface areas results from the cut off chosen for evaluation. LD surface areas tend to be larger after treatment with PA, with the difference to the control being very small. Another interesting discovery was that, at first glance, large LDs turned out to be a cluster of several smaller ones (Figure 3).

**Figure 6:**
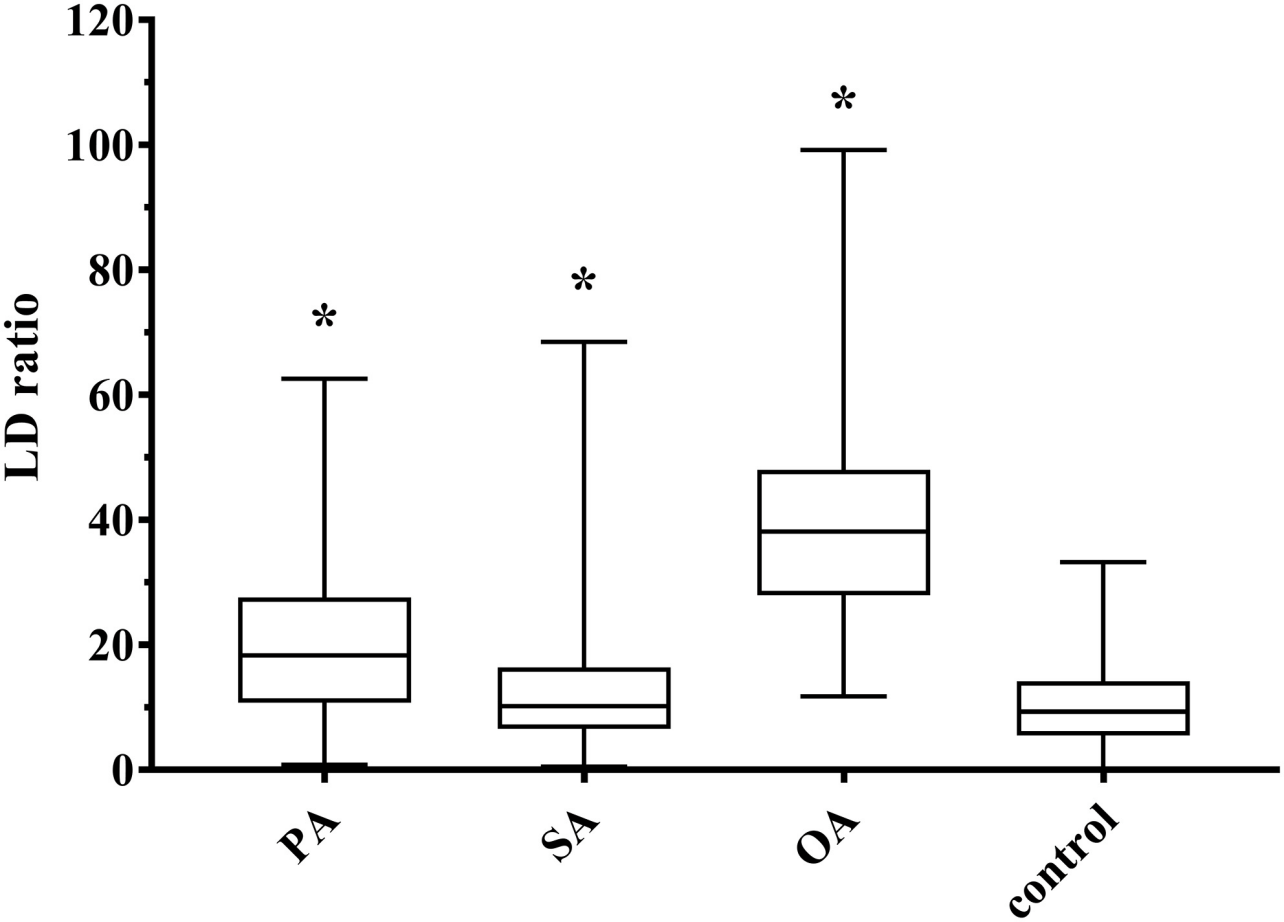
Boxplot (median; 25th to 75th percentiles; min to max) of lipid droplet (LD) ratio (LDs per cell/100 μm^2^ nucleus surface area). Asterisks represent significant difference between treatment and control (*p* < 0.05). PA, palmitic acid (*N* = 221); SA, stearic acid (*N* = 187); OA, oleic acid (*N* = 216); control (*N* = 197); *N*, the number of evaluated cells.

Larger nuclei are related with larger cells (18), which is why we assumed some relation between LDs per cell and nucleus size. In fact, we found a low positive linear correlation (*r* = 0.217; *p* < 0.001), which was even higher for single treatments, and the highest with OA (*r* = 0.548; *p* < 0.001). Consequently, we introduced the LD ratio, which is calculated by LDs per cell divided by nucleus surface area multiplied with 100. This LD ratio increased significantly after the addition of all three FA, with the highest increase after OA treatment (Figure 6). Therefore, LD ratio can be used as a measure for the ability of a FA to induce HS.

### Gene Expression in BFH12

**Figure 7:**
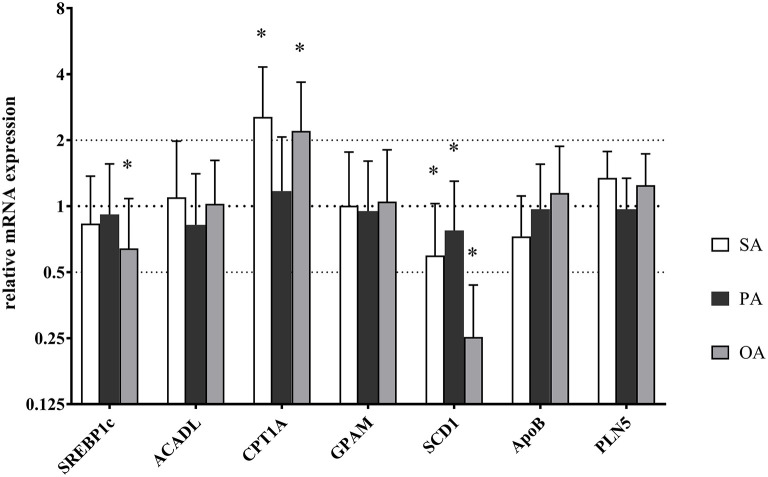
Relative mRNA expression of key enzymes in lipid metabolism after treatment with palmitic acid (PA), stearic acid (SA), or oleic acid (OA). Each value was normalized to GAPDH and SDHA expression. Error bars indicate standard error. Significant differences (*p < 0.05) between PA, SA, and OA were calculated in relation to the control (mRNA expression = 1).

RT-PCR revealed a downregulation of *SCD1* RNA expression after all three treatments in comparison to control. The addition of SA and OA showed an upregulation of *CPT1A*. OA caused a downregulation of *SREBP1c* additionally. Results are shown in Figure 7.

## Discussion

Bovine HS or so-called “fatty liver” is caused by an overload of NEFAs reaching the liver. In the first weeks of lactation, the energy demand exceeds the amount that can be obtained from the feed resulting in a NEB. This leads to a mobilization of NEFAs from adipose tissue, mainly PA and OA. This increase in plasma NEFA is followed by enhanced hepatic uptake of FA, their subsequent esterification, and accumulation of TAG in the liver (19, 20). In general, cells try to keep the level of intracellular NEFA and fatty acyl-CoA very low, by rapidly assimilating the exogenous FAs into neutral and polar lipids and oxidizing some of them (21). The studies from Gross et al. (22) have shown that NEB alone cannot cause a massive FA mobilization and TAG storage in the liver. If we follow this notion, there must be additional reasons. Ringseis et al. (23) mention stress and hormonal changes around parturition, potential infectious diseases (mastitis and endometritis), and harmful bacteria (lipopolysaccharides from gram negative bacteria) in the rumen as promotive factors.

Excessive accumulation of lipids in hepatocytes can lead to a loss of cellular integrity and function, followed by necrosis and massive liver damage (1). Our aim was to achieve lipid accumulation without loss of function. Therefore, we used non-toxic additions of PA, SA, or OA. These are the predominant FAs in ruminant plasma (24). We decided to use a model approach where the control is not based on a fatty acid-free cell treatment. Instead, we provided all cells with a basis supply of FAs, originating from FBS. The aim was to determine how BFH12 cell line responds to further addition of PA, SA, or OA on top of base FA supply already present. Thus, in our experiments, the effects obtained are smaller, but maybe closer to *in vivo* conditions. We investigated how the FA added on top influences the proportion of lipid classes and the fatty acid pattern in the lipid classes, respectively. Furthermore, LDs as hallmark of HS were evaluated with confocal laser scanning microscopy. Gene expression of key enzymes in lipid metabolism was measured in comparison to control.

### Total FA and Lipid Class Content in BFH12

Interestingly, the increase in total FA content in BFH12 after the different treatments is moderate, if at all (significant for OA only), suggesting that cells try to maintain lipid homeostasis when facing lipid overload. In this attempt, the proportions of FA within the lipid classes shift remarkably (Tables 3–5). The proportion of phospholipids, which are important membrane components, is relatively constant, while proportions of TAG increase significantly after PA and OA addition. A similar shift in total fat composition has been described for cows 1 week after calving (25). Others found a surge of PL in cows with mild fatty liver (26). When SA is added, the shift in lipid classes is little. After the addition of PA, the proportion of PL remains stable, while the proportion of TAG increases slightly and ChE decreases. In summary, FA content present in the basal medium with the addition of OA results in lipid class proportions, which are close to *in vivo* conditions. A closer look at the individual lipid classes reveals varying degrees of changes in the fatty acid pattern after each treatment.

In PL, PA was the dominant FA in the samples, and its content within PL was stable at approximately 20–30%, as described by Carta et al. (27) in humans. The same researchers (27) further describe a tight regulation of PA content in all major tissues, because of its important functions (guaranteeing membrane physical properties, protein palmitoylation, palmitoylethanolamide biosynthesis, and efficient surfactant activity in the lung). Regulation is realized *via* increased Δ9-desaturase activity (C16:0 to C16:1n7) and/or elongase (C16:0 to C18:0). Only OA and SA increase after their addition, while PA was desaturated to C16:1n7. OA was found to be the dominant FA in the cell membranes of RAW cells (3) and goose hepatocytes from overfed geese (28), which is consistent with results in BFH12 after OA treatment. Our data suggests that BFH12 prefers desaturation of PA to palmitoleic acid, although this activity is not reflected in the Δ9-desaturase index. Across all three treatments, a significant decrease in myristic acid occurs in PL, while its content increases in the TAG. This suggests a shift of C14:0, which presumably occurs in favor of membrane fluidity (29). Another unexpected discovery was the increase in Δ5-desaturase index after the addition of OA. Ballou et al. (30) described a similar but milder increase in Δ5-desaturase index after feeding fish oil to peripartal Holstein cows. The underlying mechanisms remain unclear. Overall, the calculated enzyme indices should be interpreted with caution, since on the one hand, the added product of the enzyme (OA) pass into the calculation of Δ9-desaturase index and increases the index, without increasing the activity of the enzyme. On the other hand, literature describes that the indices do not reflect the true activity in all cases (31).

Elevated TAG levels are used to characterize fatty liver in cows (1) and are seen after all three treatments in BFH12 (not significant for SA). TAGs represent the main storage form of FAs in cells. For this reason, fatty acid pattern of TAG varies greatly according to the supply of FAs. After the addition of PA, the content of saturated FAs increased markedly, together with n7 and n9 FAs. This can be explained on the one hand by the direct incorporation of PA into TAG and on the other hand by the increased Δ9-desaturase activity, which has already been discussed in a section above. Loften et al. (24) describe C16:0 as a preferred substrate for TAG over C18:0, which is consistent with our data (Table 4). SA does increase in TAG, but OA increases at almost the same rate. This, together with the increase in n7 and n9 FAs, supports the assumption that SA is preferentially desaturated to OA (32). The latter is the preferred substrate for TAG synthesis (33, 34). BFH12 show a massive incorporation of OA into TAG accompanied with a comparatively small increase in its elongation (C20:1n9) and oxidation (C16:1n9) products. Additionally, MBI increases, which fits together with the rise of n3 and n6 FA, but is contrary to the findings in humans with fatty liver (35, 36).

OA is the preferred substrate not only for TAG-synthesis but also for the acyl-CoA cholesterol acyltransferase, the enzyme responsible for ChE synthesis (33). This suggests that OA is the dominant FA in class of ChE (37). In our experiment, cholesteryl palmitate was predominant. This could be explained by the comparatively high content of PA in control. Altogether, ChEs seem to be a constant pool that awaits further use, i.e., biosynthesis and turnover of plasma lipoproteins and supplier for bile acid synthesis (37). Steadiness of ChE is reflected by our data.

As mentioned above, NEFA changes after the addition of SA only. The composition of the fatty acid pattern remains fairly stable, as well. After the addition of PA, the content of n7 and n3 FA increases, whereas the content of PA itself remains constant. This again indicates that the overall PA content is very precisely regulated (27). In general, cells try to keep the level of intracellular NEFA and fatty acyl-CoA very low, by rapidly assimilating the exogenous FAs into neutral and polar lipids, and oxidizing some of them (21). With SA treatment, the proportion of NEFA increased, which reflects that SA is not used for oxidation and TAG synthesis as readily as PA and OA. This is consistent with the findings of Pai et al. (32) in cultured rat hepatocytes, where SA was present in a greater proportion in NEFA than other FAs. Bruce et al. (34) concluded that the conversion of stearate into stearoyl-CoA may be a rate-limiting step, perhaps due to poor metabolism of stearate by the long-chain acyl-CoA synthase (38). This is one possible explanation why SA is increasingly present as a NEFA in the BFH12.

To sum up, the addition of PA, SA, or OA to the basal medium changed lipid classes and their fatty acid pattern. The addition of OA results in lipid class proportions and changes in fatty acid pattern, which are close to *in vivo* conditions. SA and PA are also able to change fatty acid pattern. However, these FAs are more lipotoxic for BFH12.

### Lipid Droplets

Another hallmark of fatty liver are LDs (6), which were visible in all BFH12 cells including control. LD ratio was introduced to overcome the influence of cell size on number of LDs per cell. LD ratio increased with addition of OA, PA, and SA. As OA is used in a wide range of models for HS, and with the massive rise of TAG, this increase is no surprise. Interestingly, PA treatment also leads to a significant increase in LD ratio and in size of LD surface areas, which supports the assumption that low doses of PA are more steatogenic and less cytotoxic (39). LDs can be divided into nascent LDs, starting from 25 nm, initial LDs (300–800 nm), expanding LDs (>1 μm), and giant LDs, which can grow up to 100 μm in adipocytes (5, 40). The smallest LD surface areas we analyzed in BFH12 showed a calculated diameter of 280 nm, referring to initial LDs. Treatment with PA leads to larger LDs in our experiments with a LD surface area of 1.83 μm^2^. Assuming a round shape, the corresponding diameter is 1.52 μm, but LDs are irregular shaped (Figure 3). With our precise method using CLSM, deconvolution, and image segmentation, we can show that, at first glance, large LDs turned out to be a cluster of several smaller ones (Figure 3). Shi et al. (41) described that in nematodes, SCD activity is necessary to build larger LDs, and this might also be true in mammalian cells, such as BFH12. Another reason why we did not see giant LDs might be the timing. Paramitha et al. (42) recently described LDs in a human hepatocellular model with FA concentrations similar to the ones we used. They found comparable LD sizes after 24 h of incubation with FA. Furthermore, they saw an increase in size after 5 days of treatment, so maybe growing of LDs into giant ones need more time.

### Gene Expression in BFH12

Continuing with gene expression, carnitine palmitoyl transferase 1, encoded by *CPT1A*, was upregulated as expected after treatment with OA and SA. The *CPT1A* regulates the uptake of NEFA by mitochondria (24), which is a main step onto β-oxidation. Du et al. (43) describe the upregulation for *CPT1A* in cows with mild fatty liver, furthermore demonstrating that PA affects hepatic gene regulation in primary hepatocytes from calf and cow. They could show that sterol regulatory binding protein 1c, encoded by *SREBP1c* gene, increased after treatment with PA. *SREBP*1c is a crucial transcriptional factor for lipid biosynthesis and it also targets enzymes of TAG synthesis (43, 44). Our data did not show any upregulation in *SREBP1c* after treatment with PA and even a mild downregulation after treatment with OA. We assume that, after 24 h, the FA substrates are metabolized or stored, and *SREBP1c* activity is no longer needed. Experiments with shorter incubation time or higher concentrations of added FA may reveal changes in gene expression.

After addition of SA, n9 FAs rose, and after treatment with PA, n7 FAs increased. This could be explained by Δ9-desaturation through stearoyl-CoA desaturase (*SCD1*). Consequently, we expected an increase of *SCD1* after PA and SA treatment, but instead, *SCD1* was downregulated. Emery et al. (45) stated that there is no *SCD1* activity in ruminant liver, whereas several researchers (31, 46, 47) found *SCD1* activity in bovine liver cells. Newer research from Da Costa et al. (48) reported desaturation of SA, but low *SCD1* gene expression levels, which is consistent with our findings. In conclusion, they observed a low *SCD1* activity and stated that hepatic desaturation activity seems to be carried out mainly by the enzymes fatty acid desaturase 1 and 2, encoded by *FADS1* and *FADS2*. In addition, Loften et al. (24) describe the inhibition of *SCD1* activity in bovine adipocytes through C16:0. This may occur similarly in hepatocytes.

Additional experiments should focus on a broader range of genes and include protein levels and enzyme activities to further support the idea of ruminant-specific pathways in the desaturation of SA and PA. Another important pathway, which needs to be investigated, is VLDL secretion and its underlying mechanisms. Low VLDL secretion is a major reason why cows are especially susceptible to HS (49). Experiments with BFH12 could reveal underlying mechanisms. In a second step, substances like rumen-protected choline, which is discussed to enhance hepatic VLDL secretion (49), can be tested with BFH12.

## Conclusion

In conclusion, the cell line BFH12 is able to incorporate, metabolize, and accumulate the added FA, which leads to typical changes in lipid class proportions and their fatty acid pattern. BFH12 with added OA acquire a steatotic phenotype with LDs, serving as reliable indicators of HS. BFH12 may serve as a useful *in vitro* model to study bovine hepatic lipid metabolism and its underlying molecular mechanisms.

## Data Availability Statement

The original contributions presented in the study are included in the article/supplementary material, further inquiries can be directed to the corresponding author/s.

## Author Contributions

KR, JK, and HF designed the studies and prepared the manuscript. KR, AN, AS, and JK performed all the experiments and analyzed the data. All authors revised the final draft of the manuscript. All authors contributed to the article and approved the submitted version.

## Conflict of Interest

The authors declare that the research was conducted in the absence of any commercial or financial relationships that could be construed as a potential conflict of interest.

## Publisher's Note

All claims expressed in this article are solely those of the authors and do not necessarily represent those of their affiliated organizations, or those of the publisher, the editors and the reviewers. Any product that may be evaluated in this article, or claim that may be made by its manufacturer, is not guaranteed or endorsed by the publisher.

## Abbreviations

ANOVA: analysis of variance
BFH12: cell line from bovine fetal hepatocytes
ChE: cholesterol ester
CLSM: confocal laser scanning microscopy
DAPI: 4′,6-diamidino-2-phenylindole
FA: fatty acid
FBS: fetal bovine serum
HS: hepatosteatosis
LD: lipid droplet
MBI: methylene bridge index
NEB: negative energy balance
NEFA: non-esterified fatty acid
OA: oleic acid
PA: palmitic acid
PCR: polymerase chain reaction
PL: phospholipid
SA: stearic acid
TAG: triglyceride
VLDL: very low-density lipoprotein
WME: Williams' medium E.
